# Supplementary material for: Characteristics of Urine Organic Acid Metabolites in Nonalcoholic Fatty Liver Disease Assessed Using Magnetic Resonance Imaging with Elastography in Korean Adults
Source: Diagnostics (Basel). 2022 May 11;12(5):1199. doi: 10.3390/diagnostics12051199 (PMC9140840; doi:10.3390/diagnostics12051199)
Supplement: Supplementary file 1 [file diagnostics-12-01199-s001.zip › diagnostics-1717947-supplementary.pdf]

Table S1. The characteristics in urine organic acid metabolites of the study participants

N = 133, intermediate metabolites in urine (mmol/mol creatinine)	
Adipate	0.659 (0.483–1.044)
Suberate	0.565 (0.368–0.821)
Ethylmalonate	2.191 (1.682–2.747)
Pyruvate	1.858 (1.020–2.956)
L-Lactate	16.95 (12.97–21.98)
β-Hydroxybutyrate	2.513 (1.207–5.453)
Citrate	413 (315–538)
cis-Aconitate	32.03 (25.64–39.53)
Isocitrate	45.53 (35.76–57.09)
α-Ketoglutarate	12.15 (6.78–18.58)
Succinate	3.421 (2.222–6.125)
Fumarate	0.288 (0.149–0.496)
Malate	0.454 (0.296–0.775)
Hydroxymethylglutarate	2.449 (1.933–3.269)
α-Ketoisovalerate	0.197 (0.093–0.295)
α-Ketoisocaproate	0.166 (0.105–0.254)
α-Keto-β-methylvalerate	0.675 (0.486–1.082)
Xanthurenate	0.294 (0.205–0.391)
β-Hydroxyisovalerate	2.379 (1.689–3.052)
Methylmalonate	1.423 (1.067–1.754)
Formiminoglutamate	0.425 (0.274–0.621)
Vanillylmandelate	1.922 (1.503–2.479)
Homovanillate	2.104 (1.542–2.938)
5-Hydroxyindoleacetate	2.213 (1.746–3.010)
Kynurenate	0.932 (0.697–1.124)
Quinolinate	2.432 (1.891–2.926)
Picolinate	0.352 (0.250–0.519)
p-Hydroxyphenyllactate	0.300 (0.219–0.453)
8-Hydroxy-2-deoxyguanosine	2.165 (1.554–2.842)
2-Methylhippurate	0.018 (0.012–0.029)
Orotate	0.635 (0.474–0.839)
Glucarate	2.886 (2.226–3.587)
α-Hydroxybutyrate	0.384 (0.241–0.631)
Pyroglutamate	18.74 (16.23–20.65)
Sulfate	2480 (1953–3165)
Benzoate	0.448 (0.346–0.644)
Hippurate	93.1 (47.3–173.8)
Phenylacetate	0.066 (0.033–0.108)
Phenylpropionate	0.008 (0.008–0.008)
p-Hydroxybenzoate	0.239 (0.149–0.371)
p-Hydroxyphenylacetate	3.615 (2.245–5.224)
Indican	14.33 (9.39–20.64)
Tricarballylate	0.242 (0.141–0.386)
D-Lactate	2.070 (0.679–4.566)
3,4-Dihydroxyphenylpropionate	0.019 (0.006–0.094)
Arabinitol	34.27 (26.35–45.08)

Data were expressed as median (interquartile range). The values of the metabolite levels were expressed using the unit of mmol/mol creatinine.

Table S2. The odds ratios of high-level metabolites for hepatic steatosis and fibrosis

	Hepatic steatosis		Hepatic fibrosis	
	OR	*P*	OR	*P*
Adipate	0.247 (0.564–1.285)	0.242	0.588 (1.311–2.921)	0.645
Suberate	0.423 (0.938–2.077)	1.000	0.690 (1.552–3.491)	0.387
Ethylmalonate	0.469 (1.028–2.254)	1.000	0.206 (0.455–1.006)	0.077
Pyruvate	0.948 (2.146–4.857)	0.100	0.504 (1.137–2.564)	0.917
L-Lactate	0.877 (1.961–4.382)	0.147	0.464 (1.033–2.302)	1.000
β-Hydroxybutyrate	0.281 (0.628–1.408)	0.351	0.206 (0.455–1.006)	0.077
Citrate	0.647 (1.415–3.093)	0.502	0.395 (0.864–1.888)	0.867
cis-Aconitate	0.356 (0.794–1.771)	0.718	0.364 (0.802–1.765)	0.728
Isocitrate	0.590 (1.300–2.863)	0.653	0.428 (0.943–2.079)	1.000
α-Ketoglutarate	0.373 (0.818–1.793)	0.762	0.427 (0.928–2.016)	1.000
Succinate	0.115 (0.292–0.737)	0.013	0.305 (0.683–1.531)	0.471
Fumarate	0.552 (1.206–2.638)	0.787	0.541 (1.190–2.621)	0.816
Malate	0.694 (1.529–3.371)	0.393	0.309 (0.682–1.501)	0.452
Hydroxymethylglutarate	0.469 (1.028–2.254)	1.000	0.867 (1.968–4.466)	0.152
α-Ketoisovalerate	0.694 (1.529–3.371)	0.393	0.502 (1.110–2.459)	0.955
α-Ketoisocaproate	0.647 (1.415–3.093)	0.502	0.337 (0.737–1.610)	0.571
α-Keto-β-methylvalerate	0.452 (1.008–2.246)	1.000	0.892 (2.073–4.818)	0.131
Xanthurenate	0.605 (1.313–2.850)	0.623	0.499 (1.086–2.367)	0.992
β-Hydroxyisovalerate	0.590 (1.300–2.863)	0.653	0.364 (0.802–1.765)	0.728
Methylmalonate	0.469 (1.028–2.254)	1.000	0.287 (0.628–1.375)	0.333
Formiminoglutamate	1.570 (3.689–8.669)	0.004	0.504 (1.137–2.564)	0.917
Vanillylmandelate	0.165 (0.390–0.923)	0.048	0.364 (0.802–1.765)	0.728
Homovanillate	0.192 (0.443–1.022)	0.083	0.867 (1.968–4.466)	0.152
5-Hydroxyindoleacetate	0.397 (0.875–1.927)	0.896	0.740 (1.657–3.712)	0.301
Kynurenate	0.647 (1.415–3.093)	0.502	0.632 (1.402–3.109)	0.527
Quinolinate	0.814 (1.800–3.980)	0.208	0.810 (1.846–4.205)	0.205
Picolinate	1.042 (2.270–4.948)	0.059	0.623 (1.360–2.966)	0.564
p-Hydroxyphenyllactate	1.316 (2.980–6.747)	0.013	0.588 (1.311–2.921)	0.645
8-Hydroxy-2-deoxyguanosine	0.440 (0.959–2.091)	1.000	0.227 (0.496–1.084)	0.115
2-Methylhippurate	0.581 (1.238–2.635)	0.719	0.713 (1.543–3.340)	0.362
Orotate	0.281 (0.628–1.408)	0.351	0.632 (1.402–3.109)	0.527
Glucarate	0.605 (1.313–2.850)	0.623	0.499 (1.086–2.367)	0.992
α-Hydroxybutyrate	0.877 (1.961–4.382)	0.147	0.464 (1.033–2.302)	1.000
Pyroglutamate	0.140 (0.340–0.828)	0.026	0.464 (1.033–2.302)	1.000
Sulfate	0.552 (1.206–2.638)	0.787	0.120 (0.273–0.625)	0.003
Benzoate	0.264 (0.590–1.316)	0.273	0.581 (1.274–2.790)	0.684
Hippurate	0.203 (0.471–1.091)	0.115	0.309 (0.682–1.501)	0.452
Phenylacetate	0.380 (0.851–1.909)	0.852	0.545 (1.222–2.739)	0.776
Phenylpropionate	0.452 (1.008–2.246)	1.000	0.394 (0.875–1.943)	0.901
p-Hydroxybenzoate	0.423 (0.938–2.077)	1.000	0.810 (1.846–4.205)	0.205
p-Hydroxyphenylacetate	0.590 (1.300–2.863)	0.653	0.221 (0.491–1.091)	0.119
Indican	0.552 (1.206–2.638)	0.787	0.395 (0.864–1.888)	0.867
Tricarballylate	0.220 (0.497–1.125)	0.135	0.427 (0.928–2.016)	1.000
D-Lactate	0.356 (0.794–1.771)	0.718	0.588 (1.311–2.921)	0.645
3,4-Dihydroxyphenylpropionate	0.247 (0.564–1.285)	0.242	0.810 (1.846–4.205)	0.205
Arabinitol	0.647 (1.415–3.093)	0.502	0.867 (1.968–4.466)	0.152

High level is defined as the fourth quartile in each metabolite. *P* values were calculated using chi-square tests.
